# 

**Published:** 1874-02

**Authors:** 


### CONTENTS.

### COMMUNICATIONS.

Contour Fillings.... 53

Sensitive Dentine and Its Remedy.... 59

Address.... 64

A New Method of Pivoting.... 68

Professional Pride.... 69

Diagnosis.... 71

### SELECTIONS.

The Diarrhœa of Teething Children.... 72

Oatmeal, Bone and Muscle.... 73

The Process of Enbalming.... 74

### PROCEEDINGS OF SOCIETIES.

Minutes of the Ohio State Dental Society.... 75

### EDITORIAL.

Book Review.... 88

Vulcanite Litigation.... 90

Gold Caps.... 92

Automatic Saliva Ejector.... 93

Popular Instruction.... 94

Journalistic.... 95

Mississippi Valley Dental Society.... 95

Ohio Dental College Association.... 96

Commencement.... 96

